# Supplementary material for: The power and promise of genetic mapping from Plasmodium falciparum crosses utilizing human liver-chimeric mice
Source: Commun Biol. 2021 Jun 14;4:734. doi: 10.1038/s42003-021-02210-1 (PMC8203791; doi:10.1038/s42003-021-02210-1)
Supplement: Supplementary file 2 — Supplemental Information [file 42003_2021_2210_MOESM2_ESM.pdf]

The power and promise of genetic mapping from *Plasmodium falciparum* crosses utilizing human liver-chimeric mice

Katrina A. Button-Simons[1*], Sudhir Kumar[2], Nelly Carmago[2], Meseret T. Haile[2], Catherine Jett[3], Lisa A. Checkley[1], Spencer Y. Kennedy[2], Richard S. Pinapati[4], Douglas A. Shoue[1], Marina McDew-White[5], Xue Li[5], François H. Nosten[6,7], Stefan H. Kappe[2], Timothy J. C. Anderson[5], Jeanne Romero-Severson[8], Michael T. Ferdig[1], Scott J. Emrich[9], Ashley M. Vaughan[2], Ian H. Cheeseman[3*]

[1]*Eck Institute for Global Health, Department of Biological Sciences, University of Notre Dame, Notre Dame, IN, USA*
[2]*Center for Global Infectious Disease Research, Seattle Children's Research Institute, Seattle, WA, USA*
[3]*Host Pathogen Interactions Program, Texas Biomedical Research Institute, San Antonio, TX, USA*
[4]*Nimble Therapeutics, Madison, WI*
[5]*Disease Intervention and Prevention Program, Texas Biomedical Research Institute, San Antonio, TX, USA*
[6]*Shoklo Malaria Research Unit, Mahidol-Oxford Tropical Medicine Research Unit, Mahidol University, Mae Sot, Thailand*
[7]*Centre for Tropical Medicine and Global Health, Nuffield Department of Medicine Research building, University of Oxford Old Road campus, Oxford, UK*
[8]*Department of Biological Sciences, University of Notre Dame, IN*
[9]*Univeristy of Tennessee, Knoxville, TN, USA*

* Corresponding author

Supplemental Figures and Tables

Supplemental Table 1

Cross	Cage	Post feed exflagellation	Number of Mosquitos	Prevalence	Oocysts/Mosquito Mean (Median)	Mouse infection method	Parasitemia after exsanguination
NF54WT x NHP4026	NHP4026 control	4.9	250	56%	3.4 (1)	IV	0.047%
NF54WT x NHP4026	NF54 control	3.4	250	45%	9.2 (0.5)	MB	0.110%
NF54WT x NHP4026	pooled	4.9	250	67%	7.9 (3)	none	NA
NF54WT x NHP4026	pooled	4.9	250	91%	18.3 (5)	none	NA
NF54WT x NHP4026	pooled	4.9	250	73%	8.4 (3)	MB	0.017%
NF54WT x NHP4026	pooled	4.9	250	73%	6 (2)	IV	0.013%
NF54WT x NHP4026	pooled	4.9	250	58%	22.4 (3)	MB	0.020%
NF54WT x NHP4026	pooled	4.9	250	70%	19.9 (17)	none	NA
MKK2835 x NHP1337	MKK2835 control	2	200	62.5	1.5 (2)	none	NA
MKK2835 x NHP1337	NHP1337 control	1.5	200	77	2 (2)	none	NA
MKK2835 x NHP1337	pooled	1.57	202	30	0.6 (0)	none	NA
MKK2835 x NHP1337	pooled		204	80	3 (3)	IV	4.500%
MKK2835 x NHP1337	MKK2835 control	1	200	37.5	0.87 (0)	none	NA
MKK2835 x NHP1337	NHP1337 control	0.42	200	33.3	0.77 (0)	none	NA
MKK2835 x NHP1337	pooled	0.66	200	54	2 (2)	none	NA
MKK2835 x NHP1337	pooled		200	60	1.1 (1)	none	NA

Mosquito feed data and parasitemia at mouse exsanguination for NF54 x NHP4026 cross and MKK2835 x NHP1337 cross. Anopheles Stephensi mosquitoes in a given cage were fed blood containing either both parents of the cross (pooled) or a single parents as a control. The post feed exflagelation is the number of exflaggelation events per field of view. The number of mosquitos is the number of mosquitos harvested per cage. The prevalence is the percent of mosquitos dissected with oocysts present in the midgut. The Oocysts/Infected Mosquito is the average and mean number of oocysts per infected mosquito midgut. Mouse infection method provides information on which cages were used to infect mice either by intrevenous (IV) injection or mosquito bite (MB),

other cages were not used to infect mosquitos (none). The parasitemia after exsanguination column provides the parasitemia of blood recovered immediately after mouse exsanguination.

Supplemental Table 2

Cross/Cloning	Days before cloning	Source	Cloning Media	Cloned Progeny (Genotyped Progeny)	Selfed Progeny	Non-clonal Progeny	Repeat Genotypes within (between) cloning rounds	Unique Recombinant Progeny
NF54 x NHP4026				175 (168)	3	25	55 (1)	84
NF54HT-GFP–luc x NHP4026: Cloning 1	0	straight from mouse	CM w/ serum	27 (27)	3	1	3 (0)	20
NF54HT-GFP–luc x NHP4026: Cloning 2	14	cryopreserved bulk population	CM w/ serum	37 (37)	0	2	23 (0)	12
NF54HT-GFP–luc x NHP4026: Cloning 3	19	cryopreserved bulk population	CM w/ albumax	60 (60)	0	5	29 (1)	25
NF54WT x NHP4026: Cloning 1	5	straight from mouse	CM w/ serum	51 (46)	0	17	0 (0)	27
MKK2835 x NHP1337				266 (248)	149	36	3 (0)	60
MKK2835 x NHP1337: Cloning 1	5	straight from mouse	CM w/ albumax	74 (74)	45	11	0 (0)	18
MKK2835 x NHP1337: Cloning 2	2	cryopreserved bulk population	CM w/ albumax	192 (174)	104	25	3 (0)	42

Progeny characterization by cloning round. Total number of genotyped progeny are given along with their characterization as selfed progeny, non-clonal progeny, sampling of repeat genotypes and unique recombinant progeny for each cross and broken down by each cloning round. Days before cloning denotes the number of days from mouse exsanguination and/or thaw of cryopreserved stocks before cloning was initiated. Source denotes whether cloning was initiated from coninuous culture after mouse exsanguination and transition to in vitro culture (straight from mouse) or whether transitioned in vitro culture was cryopreserved and then thawed and cloning initiated after the thaw (cryopreserved bulk population). Cloning Media is either complete media used to standard Plasmodium falciparum culture with albumax (CM w/ albumax) or human serum (CM w/ serum)

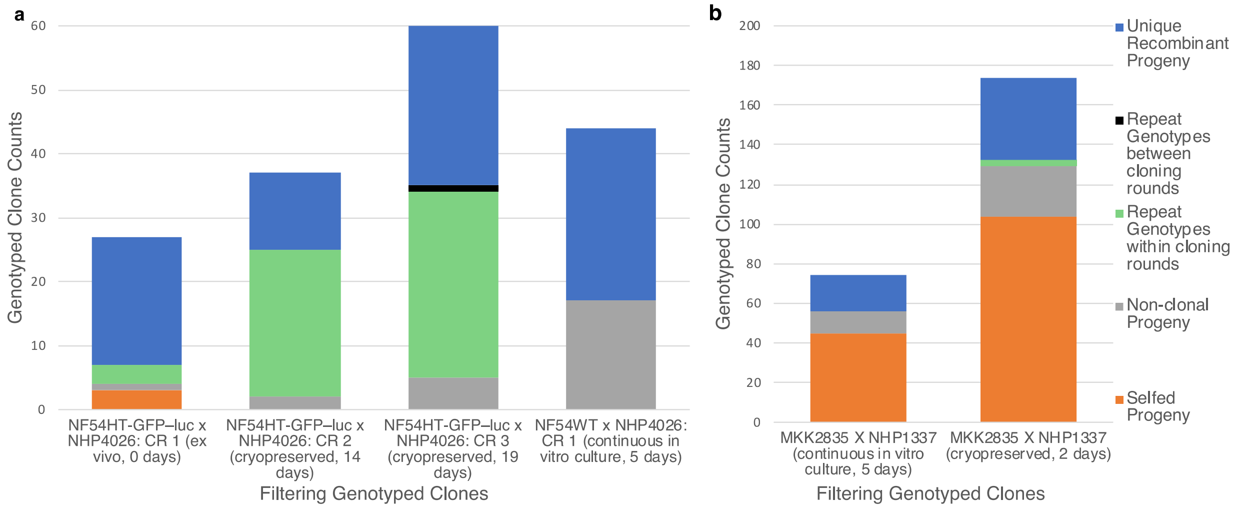

Supplemental Figure 1. Cloning results for each cross by biological replicate and cloning round. Progeny for the NF54 × NHP4026 cross (a) and the MKK2835 × NHP1337 cross (b) were filtered to identify unique recombinant progeny (blue). Selfed progeny (orange), non-clonal progeny (grey) and repeat sampling of the same genotype within a cloning round (green) and between cloning rounds (black) were filtered out of total genotyped progeny for each biological replicate and cloning round. The proportion of unique recombinant progeny recovered decreased substantially when cloning was initiated after 14 to 19 days of *in vitro* culture.

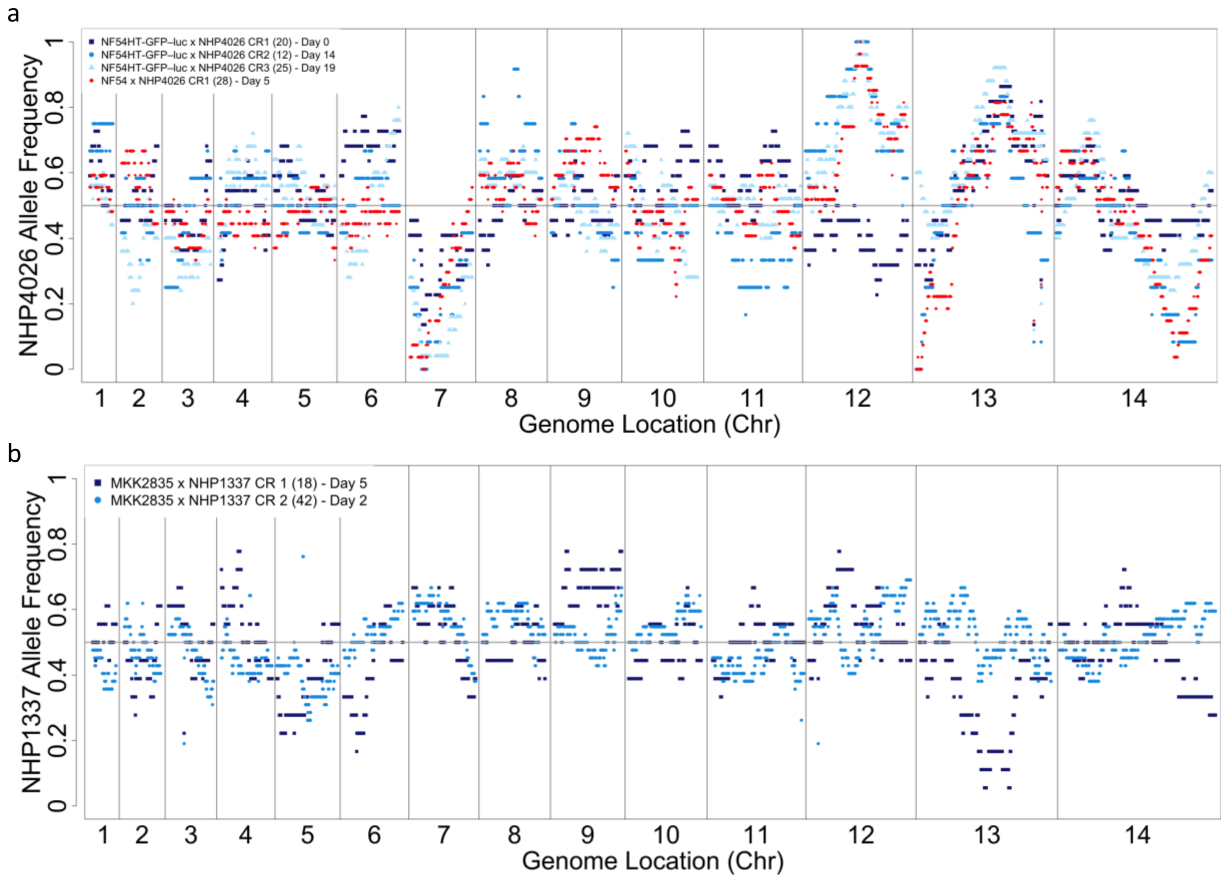

Supplemental Figure 2. Genome-wide allele frequency in each cross by cloning round. Allele frequencies for unique recombinant progeny for the NF54xNHP4026 cross (a) and MKK2835xNHP1337 cross (b) plotted across the genome for progeny from each cloning round. The day after exsanguination that each cloning round was initiated is listed in the legend.

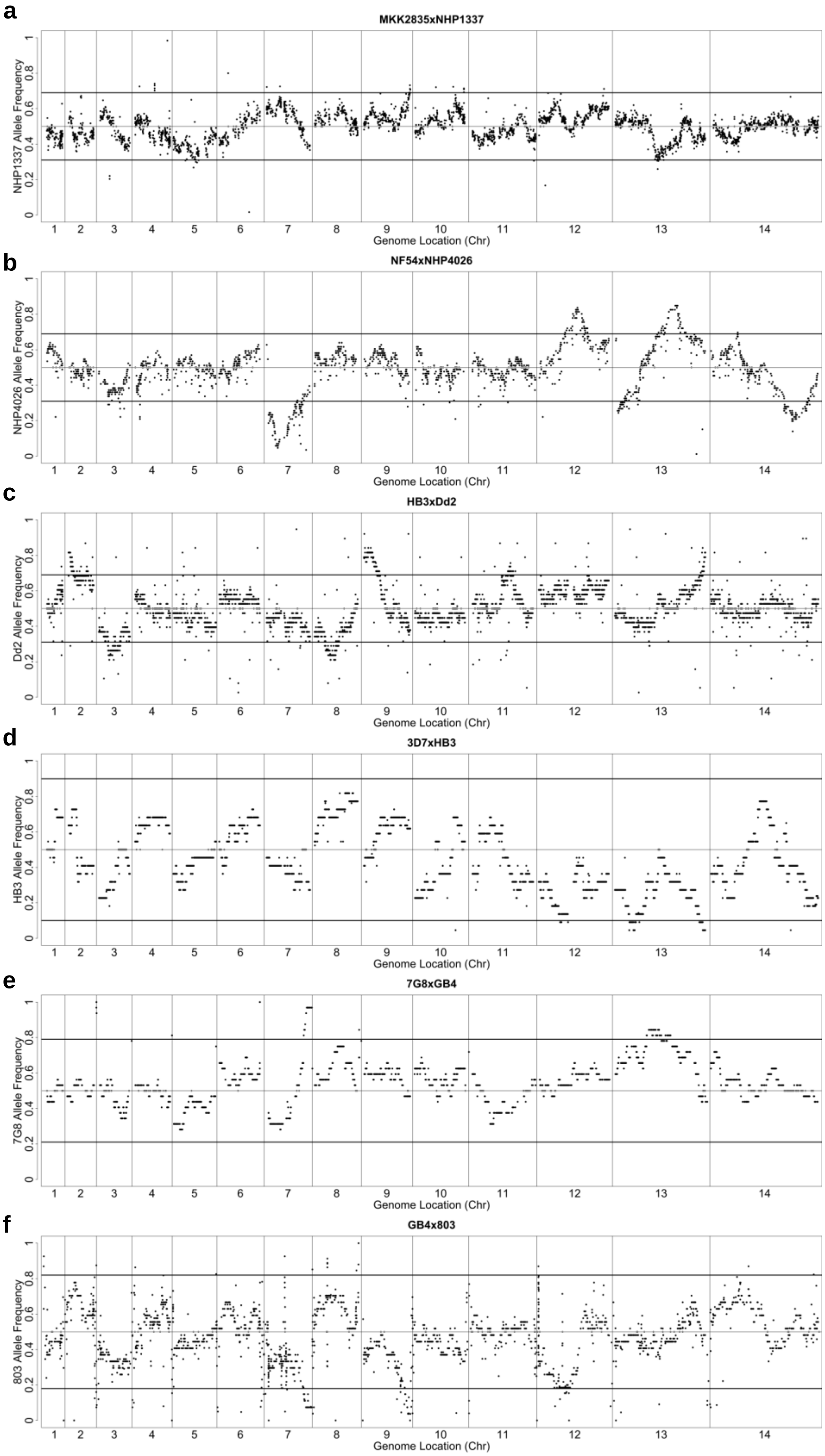
a
MKK2835xNHP1337
NHP1337 Allele Frequency
b
NF54xNHP4026
NHP4026 Allele Frequency
c
HB3xDd2
Dd2 Allele Frequency
d
3D7xHB3
HB3 Allele Frequency
e
7G8xGB4
7G8 Allele Frequency
f
GB4x803
803 Allele Frequency
Genome Location (Chr)

Supplemental Figure 3. Segregation distortion in unique recombinant progeny from all published *P. falciparum* crosses. Allele frequencies for unique recombinant progeny plotted across the genome for all six published *P. falciparum* crosses[1,2] show no significant segregation distortion in the MKK2835xNHP1337 cross (a) in contrast to all other published crosses which show regions of significant segregation distortion (b-f).

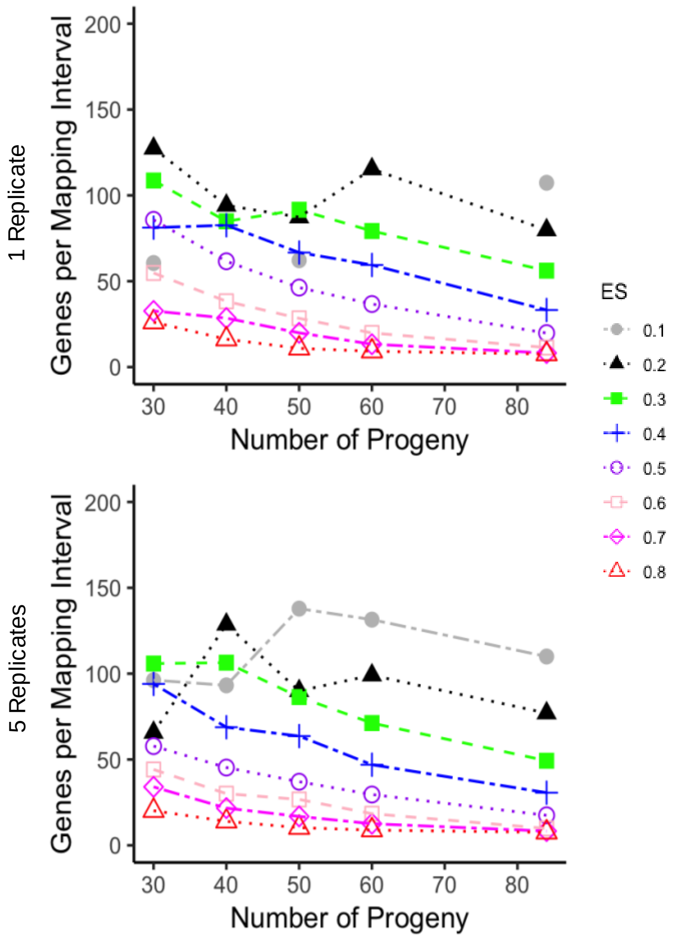

Supplemental Figure 4. Mapping Resolution for different size progeny sets. Average mapping resolution reported as number of genes per 1.5 LOD interval for simulated phenotypes that accurately map to the 1.5 LOD interval surrounding the causal loci. Progeny set size varied from the full NF54 × NHP4026 progeny set of 84 and was subsampled at 30, 40, 50, 60 and 70 progeny. Each curve represents phenotypes simulated with a given effect size (ES) with ES ranging between 0.1 to 0.8.

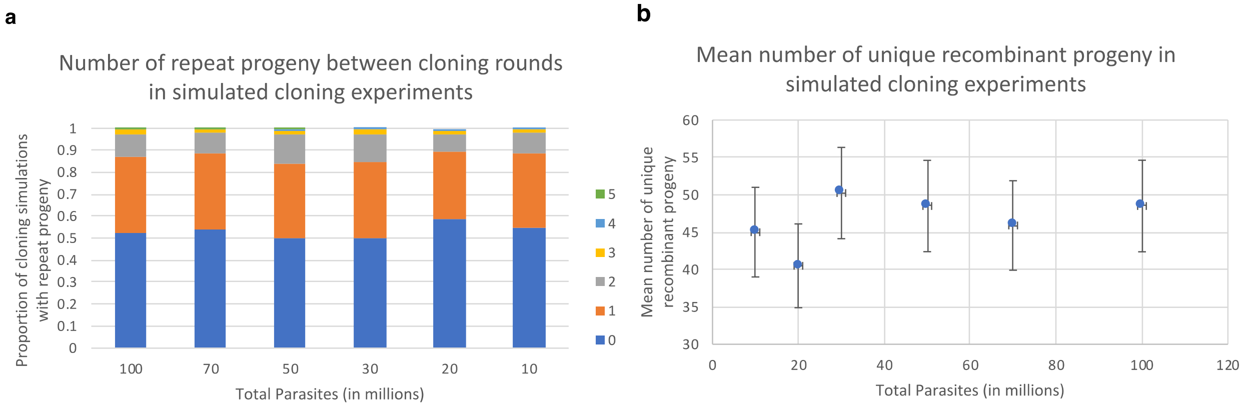

Supplemental Figure 5 Simulation of sampling across cloning rounds. We performed a simulation analysis to determine how often we might expect to observe little to no repeat sampling between cloning rounds. We simulated sampling from different culture sizes and parasitemias using observed oocysts per infected mosquito and the percentages of selfed progeny, and unique recombinants from the MKK2835 × NHP1337 cross. We assumed each unique genotype is represented 1000x to 30000x in the total population of 10,000,000 to 100,000,000 parasites and performed random draws of genotypes. For each set of 1000 cloning simulations we calculated the proportion of simulations with different amounts of repeat sampling (a) and the mean number of unique recombinant progeny (b). Across our range of total parasites the unique recombinant progeny ranges from 40 to 50 with 80%-90% of simulated cloning rounds having zero or one progeny samples in both cloning rounds.

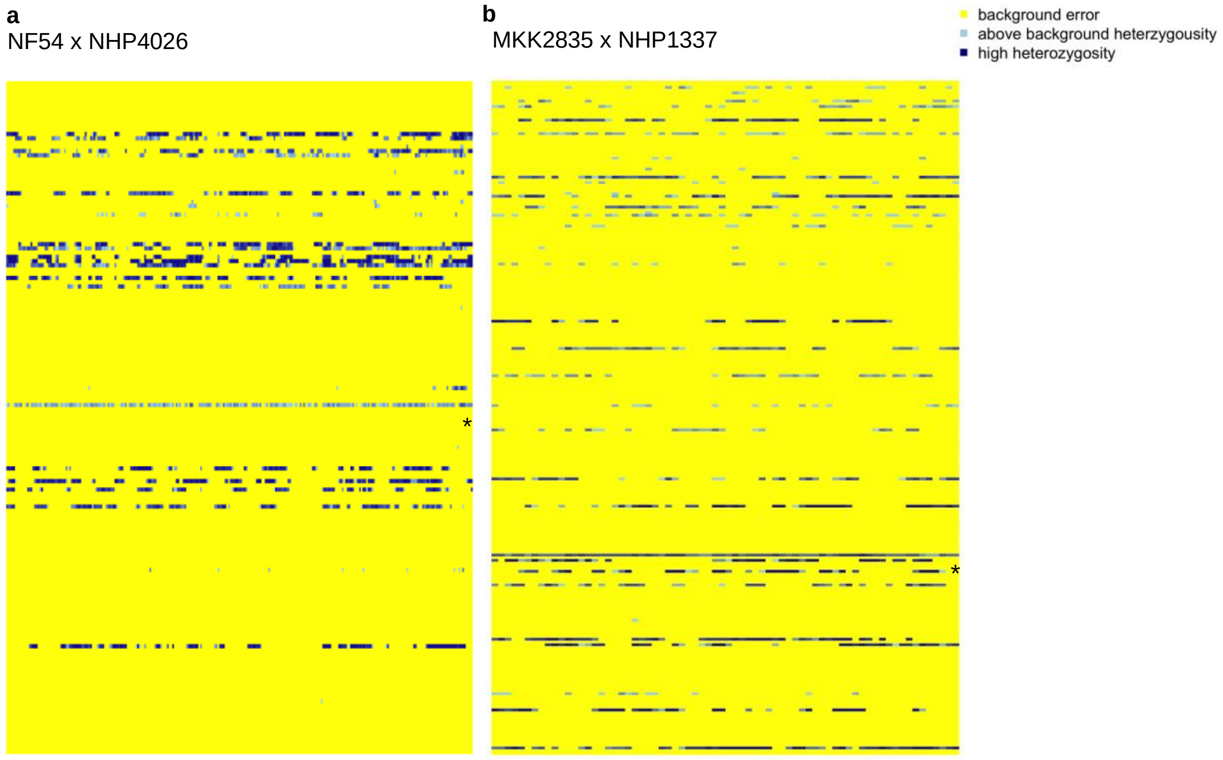

Supplemental Figure 6. Nonclonal Progeny Heatmap. Heatmap showing regions of the genome for each progeny with above expected numbers of heterozygous allele calls. Regions with above expected heterozygous SNP calls were identified through a sliding window analysis. Progeny along with an uncloned sample (denoted with an *) are shown as rows and each column represents a 90kb region (window size was defined as the expected distance between heterozygous SNP calls based on the heterozygous SNP call rate for each cross). (a) In progeny of the NF54 × NHP4026 cross, 25 progeny had regions with above expected heterozygosity. (b) In progeny of the MKK2835 × NHP1337 cross, 35 progeny had regions with above expected heterozygosity. The un-cloned samples (*) show above background heterozygosity or high heterozygosity across the genome.

Methods

Cloning Protocol: Cloning from F_1 progeny populations of *Plasmodium falciparum* genetic crosses by limiting dilution

The following procotol is a modification of previously published protocols for cloning by limiting dilution[3]. The use of the Phusion Blood Direct PCR Kit has allowed us to detect positives using only 3 µL of culture which has greatly expanded our ability to clone. In earlier cloning rounds we were only able to handle 60-100 clones at once. In recent cloning rounds we have successfully expanded 560 clones in a single cloning round.

1) Cloning plate set up:

<u>Preparation of Initial Culture</u>

Cloning can be initiated directly after *in vitro* transition after mouse exsanguination or from cryopreserved stocks. Cryopreserved parasite stocks should be thawed into complete media containing RPMI 1640 with L-glutamine (Invitrogen Corp.), 50 mg/L hypoxanthine (Sigma-Aldrich), 25 mM HEPES (Cal Biochem), 0.5% Albumax II (Invitrogen Corp.), 10 mg/L gentamicin (Invitrogen Corp.) and 0.225% $NaHCO_3$ (Biosource) at 5% hematocrit using fresh human RBCs. Maintain cultures for 2 complete cycles at 37˚C under an atmosphere of 5% CO_2, 5% O_2, and 90% N_2 with gentle shaking. Prepare a thin smear and count parasitemia. Expand culture aggressively such that invasion occurs when parasitemias are below 1%. In order to maximize unique recombinant progeny recovered initiate cloning as soon as possible after transition to *in vitro* culture.

<u>Cloning Plate dilutions</u>

Set up plates as follows for 0.25 parasites/well:

Dilution 1:

Volume of complete media = %Parasitemia/2

Volume of culture = 10 µL culture

Dilute 10 µL culture in the calculated amount of complete media and mix well. For example: for a culture at 1% parasitemia, add 500 uL of CM to 10 uL of culture.

Dilution 2: Add 10 µL of Dilution 1 to 1 mL of complete media in a tube and mix well.

Dilution 3: Add 200 µL of Dilution 2 to 38mLs of complete media + 2 mLs of RBC suspension (1:1 RBC to incomplete media, use fresh RBCs). Mix well.

Plate 200 uL of the above dilution into 95 wells of a 96 well plate with a mulit-channel pipette using a new set of tips for each row, leaving well A1 empty. Fill well A1 with RBCs and complete media. Put the plates into a clean chamber with a 96 well plate of H_2O in the bottom for humidity or a gassed incubator at an atmosphere of 5% CO_2, 5% O_2, and 90% N_2.

*To maximize returns we can use a larger amount of original culture and go as high as 0.5 parasites/well. At this level we have observed 10-20% of positive wells are non-clonal. We can then sub-clone to get additional parasites.

<u>Cloning Plate Maintenance</u>

Change media on plates weekly by removing 150 µL of media (use new tips for each row- one box of tips per plate) and replace with 150 µL s of a mixture of 30 mLs complete media + 400 µL s of RBCs that has been mixed well. As plates age if media begins to look dark and RBC lysis increases, change the media more frequently (every 4-5 days) to ensure parasites have a fresh supply of RBCs.

Gas the chambers every 2 days.

While changing media on plates, clean the chamber out with 10% bleach water and also clean the plate with the water in the bottom of the chamber. Refill the plate for humidity and put it and the clean disassembled chamber into a hood and UV it for 30 mins.

2) Screening cloning plates:

kit for PCR: **Phusion Blood Direct PCR Kit Thermo Scientific cat# F-547L**. Use the specified primer calculator for specific TM for this kit.

Invitrogen/Life Sciences/Molecular Probes SYBR Green I nucleic acid stain 10,000X cat # S7585. Dilute this in water to ~3X concentration (since once it goes into reaction will be ~1X in a tube and use that as water for reactions).

Primers – Can use any primers with a small amplicon (<100 bp) that are well behaved. We recommend either the microsatellite primer for PE14D with an annealing temp of 54°.

Master Mix

1RXN:		500 RXN Master mix:
2X buffer	5.0 uL	2500 uL
enzyme	0.1 uL	50 uL
F. Primer	0.25 uL	125 uL
R. Primer	0.25 uL	125 uL
H_2O (w/SYBR)	1.4 uL	700 uL
	7.0 uL	3500 uL

Test Plate

-add 7 uL of the master mix to the test plate
-dilute whole culture 1:4 (1 uL of whole culture from cloning plates into 3 uL of water) and add 3 uL of diluted to the test plate (do this right after media changes so the wells are already mixed up).
-seal the plate and run on the qPCR protocol

PCR Protocol

- ABI 7900HT for pfCRT primers

stage 1=95° for 20 seconds
stage 2=95° for 1 second
62.3° for 30 seconds
65° for 15 seconds
(stage 2: 30 cycles)

-set to "fast" mode, reaction volume= 10 uL, and "SYBR detector no quencher"
-plate run time is between 30-40 mins
-after the run and analysis use the CT score to determine positives. We have found that pulling cultures from wells with CT score below 21 and expanding these to 1 mL gives good success. Because the kit is so sensitive it is possible to leave questionable wells and screen them again the next week. A standard curve of parasitemia to CT score is provided in Davis et al.[4] for comparison of parasitemia.

Positives are pulled to 1 mL wells at 5% Hct in 24 well plates and maintained at standard culture conditions to generate stock pellets and pellets for DNA isolation.

Stock Pellets: Parasites need to be greater than 1% parasitemia and mostly ring.
DNA Pellets: Let the wells grow up until the parasitemia is above 3% and late stage then freeze the whole well/culture down for DNA.

References

1 Miles, A. *et al.* Indels, structural variation, and recombination drive genomic diversity in Plasmodium falciparum. *Genome research* **26**, 1288-1299, doi:10.1101/gr.203711.115 (2016).

2 Sá, J. M. *et al.* Artemisinin resistance phenotypes and K13 inheritance in a Plasmodium falciparum cross and Aotus model. *Proceedings of the National Academy of Sciences*, doi:10.1073/pnas.1813386115 (2018).

3 Butterworth, A. S. *et al.* An improved method for undertaking limiting dilution assays for in vitro cloning of Plasmodium falciparum parasites. *Malaria Journal* **10**, doi:10.1186/1475-2875-10-95 (2011).

4 Davis, S. Z. *et al.* The extended recovery ring stage survival assay provides superior prediction of patient clearance half life and increases throughput. *bioRxiv*, doi:10.1101/846329 (2019).
