# Supplementary material for: The power and promise of genetic mapping from Plasmodium falciparum crosses utilizing human liver-chimeric mice
Source: Commun Biol. 2021 Jun 14;4:734. doi: 10.1038/s42003-021-02210-1 (PMC8203791; doi:10.1038/s42003-021-02210-1)
Supplement: Supplementary file 3 — Description of Additional Supplementary Files [file 42003_2021_2210_MOESM3_ESM.pdf]

Description of Additional Supplementary Files

File Name: Supplementary Data 1
Description: NF54/NF54HT-GFP–luc × NHP4026 Genetic Map

File Name: Supplementary Data 2
Description: MKK2835 × NHP1337 Genetic Map

File Name: Supplementary Data 3
Description: Allele frequencies and significance of segregation distortion in NF54 × NHP4026 progeny.

File Name: Supplementary Data 4
Description: Microsatelite information for microsatellites used for initial screening of some of the NF54 × NHP4026 progeny.
