# Supplementary material for: The power and promise of genetic mapping from Plasmodium falciparum crosses utilizing human liver-chimeric mice
Source: Commun Biol. 2021 Jun 14;4:734. doi: 10.1038/s42003-021-02210-1 (PMC8203791; doi:10.1038/s42003-021-02210-1)
Supplement: Supplementary file 8 — Reporting Summary [file 42003_2021_2210_MOESM8_ESM.pdf]

nature research

Corresponding author(s): Katrina A Button-Simons, Ian H. Cheeseman

Last updated by author(s): 3/9/2021

Reporting Summary

Nature Research wishes to improve the reproducibility of the work that we publish. This form provides structure for consistency and transparency in reporting. For further information on Nature Research policies, see our Editorial Policies and the Editorial Policy Checklist.

Statistics

For all statistical analyses, confirm that the following items are present in the figure legend, table legend, main text, or Methods section.

n/a	Confirmed	
☐	☒	The exact sample size (*n*) for each experimental group/condition, given as a discrete number and unit of measurement
☐	☒	A statement on whether measurements were taken from distinct samples or whether the same sample was measured repeatedly
☐	☒	The statistical test(s) used AND whether they are one- or two-sided<br>*Only common tests should be described solely by name; describe more complex techniques in the Methods section.*
☒	☐	A description of all covariates tested
☐	☒	A description of any assumptions or corrections, such as tests of normality and adjustment for multiple comparisons
☐	☒	A full description of the statistical parameters including central tendency (e.g. means) or other basic estimates (e.g. regression coefficient) AND variation (e.g. standard deviation) or associated estimates of uncertainty (e.g. confidence intervals)
☐	☒	For null hypothesis testing, the test statistic (e.g. F, t, r) with confidence intervals, effect sizes, degrees of freedom and P value noted<br>*Give P values as exact values whenever suitable.*
☒	☐	For Bayesian analysis, information on the choice of priors and Markov chain Monte Carlo settings
☒	☐	For hierarchical and complex designs, identification of the appropriate level for tests and full reporting of outcomes
☐	☒	Estimates of effect sizes (e.g. Cohen's *d*, Pearson's *r*), indicating how they were calculated

Our web collection on statistics for biologists contains articles on many of the points above.

Software and code

Policy information about availability of computer code

Data collection	Variant calling for sequencing<br>We aligned raw sequencing reads to v3 of the 3D7 genome reference (http://www.plasmodb.org) using BWA MEM v0.7.5a. After removing PCR duplicates and reads mapping to the ends of chromosomes (Picard v1.56) we recalibrated base quality scores, realigned around indels and called genotypes using GATK v3.5 in the GenotypeGVCFs mode using QualByDepth, FisherStrand, StrandOddsRatio VariantType, GC Content and max_alterate_alleles set to 6. We recalibrated quality scores and calculated VQSLOD scores using SNP calls conforming to Mendelian inheritance in previous genetic crosses, and excluding sites in highly error-prone genomic regions (calls outside of the “core genome” as defined in Miles et al [doi:10.1101/gr.203711.115]).
Data analysis	After vcf files were generated all additional analysis to generate sets of informative genotype markers for clonal unique recombinants for genetic map construction is available on github https://github.com/kbuttons/P01_ProgenyCharacterization<br>Genetic maps were constructed using JoinMapv4.1

For manuscripts utilizing custom algorithms or software that are central to the research but not yet described in published literature, software must be made available to editors and reviewers. We strongly encourage code deposition in a community repository (e.g. GitHub). See the Nature Research guidelines for submitting code & software for further information.

Data

Policy information about availability of data

All manuscripts must include a data availability statement. This statement should provide the following information, where applicable:

- Accession codes, unique identifiers, or web links for publicly available datasets
- A list of figures that have associated raw data
- A description of any restrictions on data availability

All raw sequencing data have been submitted to the NCBI Sequence Read Archive (SRA, https://www.ncbi.nlm.nih.gov/sra) under the project number of PRJNA524855 as SRR11835641 to SRR11850234. VCF files and all analysis is available at https://github.com/kbuttons/P01_ProgenyCharacterization.

Field-specific reporting

Please select the one below that is the best fit for your research. If you are not sure, read the appropriate sections before making your selection.

☐ Life sciences ☐ Behavioural & social sciences ☒ Ecological, evolutionary & environmental sciences

For a reference copy of the document with all sections, see nature.com/documents/nr-reporting-summary-flat.pdf

Ecological, evolutionary & environmental sciences study design

All studies must disclose on these points even when the disclosure is negative.

Study description	Creating two new genetic crosses for Plasmodium falciparum using recently isolated patient-derived parasites - 1) an allopatric cross between a laboratory-adapted parasite (NF54) of African origin and a recently patient-derived Asian parasite, and (2) a sympatric cross between two recently derived patient-derived Asian parasites.
Research sample	All progeny cloned from two new genetic crosses.
Sampling strategy	Cloning was performed via sampling by limiting dilution immediately after transition to in vitro culture and after thawing of cryo-preserved bulk transitioned in vitro culture.
Data collection	*Describe the data collection procedure, including who recorded the data and how.*
Timing and spatial scale	Cloning plates were monitored for positive wells beginning 2 weeks after culture and continuing until no new positives were identified on two consecutive screens (6-8 weeks).
Data exclusions	Samples were excluded if they had missing data at more than 80% of the high quality parental SNPs.
Reproducibility	The sympatric cross was performed twice with highly repeatable results.
Randomization	As progeny were identified from cloning they were assigned alphanumeric codes and all sequencing was done randomly in batches.
Blinding	N/A

Did the study involve field work? ☐ Yes ☒ No

Reporting for specific materials, systems and methods

We require information from authors about some types of materials, experimental systems and methods used in many studies. Here, indicate whether each material, system or method listed is relevant to your study. If you are not sure if a list item applies to your research, read the appropriate section before selecting a response.

Materials & experimental systems

n/a	Involved in the study	
☒	☐	Antibodies
☐	☒	Eukaryotic cell lines
☒	☐	Palaeontology and archaeology
☐	☒	Animals and other organisms
☒	☐	Human research participants
☒	☐	Clinical data
☒	☐	Dual use research of concern

Methods

n/a	Involved in the study	
☒	☐	ChIP-seq
☒	☐	Flow cytometry
☒	☐	MRI-based neuroimaging

Eukaryotic cell lines

Policy information about cell lines

Cell line source(s)	Isolates used as parental lines were originally obtained during treatment of malaria infected patients visiting the Shoklo Malaria research Unit clinics on the Thailland-Myanmar border [DOI: 10.1186/s12936-019-2934-4].
Authentication	Genotype information was recorded as parasites were isolated. After lab adapting and generating individuals clones, parasites were genotyped to confirm matching genotypes between clones and original isolates. A panel of microsatelite markers were developed for quick genotyping to confirm the identity of parents and progeny for future phenotyping.
Mycoplasma contamination	N/A
Commonly misidentified lines (See ICLAC register)	N/A

Animals and other organisms

Policy information about studies involving animals; ARRIVE guidelines recommended for reporting animal research

Laboratory animals	FRG NOD huHep mouse
Wild animals	N/A
Field-collected samples	N/A
Ethics oversight	Seattle Children's Research Institute (SCRI) IACUC

Note that full information on the approval of the study protocol must also be provided in the manuscript.
